# Effects of SGLT2 Inhibition in Human Kidney Proximal Tubular Cells—Renoprotection in Diabetic Nephropathy?

**DOI:** 10.1371/journal.pone.0054442

**Published:** 2013-02-04

**Authors:** Usha Panchapakesan, Kate Pegg, Simon Gross, Muralikrishna Gangadharan Komala, Harshini Mudaliar, Josephine Forbes, Carol Pollock, Amanda Mather

**Affiliations:** 1 Department of Medicine, The University of Sydney, Renal Research Group, Kolling Institute of Medical Research, Royal North Shore Hospital, St. Leonards, New South Wales, Australia; 2 Glycation and Diabetes Research Group, Mater Medical Research Institute, South Brisbane Qld, Australia; University of Leicester, United Kingdom

## Abstract

Sodium/glucose cotransporter 2 (SGLT2) inhibitors are oral hypoglycemic agents used to treat patients with diabetes mellitus. SGLT2 inhibitors block reabsorption of filtered glucose by inhibiting SGLT2, the primary glucose transporter in the proximal tubular cell (PTC), leading to glycosuria and lowering of serum glucose. We examined the renoprotective effects of the SGLT2 inhibitor empagliflozin to determine whether blocking glucose entry into the kidney PTCs reduced the inflammatory and fibrotic responses of the cell to high glucose. We used an *in vitro* model of human PTCs. HK2 cells (human kidney PTC line) were exposed to control 5 mM, high glucose (HG) 30 mM or the profibrotic cytokine transforming growth factor beta (TGFβ1; 0.5 ng/ml) in the presence and absence of empagliflozin for up to 72 h. SGLT1 and 2 expression and various inflammatory/fibrotic markers were assessed. A chromatin immunoprecipitation assay was used to determine the binding of phosphorylated smad3 to the promoter region of the SGLT2 gene. Our data showed that TGFβ1 but not HG increased SGLT2 expression and this occurred via phosphorylated smad3. HG induced expression of Toll-like receptor-4, increased nuclear deoxyribonucleic acid binding for nuclear factor kappa B (NF-κB) and activator protein 1, induced collagen IV expression as well as interleukin-6 secretion all of which were attenuated with empagliflozin. Empagliflozin did not reduce high mobility group box protein 1 induced NF-κB suggesting that its effect is specifically related to a reduction in glycotoxicity. SGLT1 and GLUT2 expression was not significantly altered with HG or empagliflozin. In conclusion, empagliflozin reduces HG induced inflammatory and fibrotic markers by blocking glucose transport and did not induce a compensatory increase in SGLT1/GLUT2 expression. Although HG itself does not regulate SGLT2 expression in our model, TGFβ increases SGLT2 expression through phosphorylated smad3.

## Introduction

Diabetic nephropathy is the leading cause of end stage kidney disease and its escalating incidence is a challenge to health systems in both the developed and developing worlds. Treatment options have increased substantially over the last decade but nevertheless have not translated into a reduction in the incidence of end stage kidney disease related to diabetic nephropathy [1], outlining the continuing need for agents that have a specific effect on the renal disease associated with diabetes.

Sodium/glucose co-transporter 2 inhibitors (SGLT2inh) are promising (not yet on the market) agents used to achieve glycaemic control in type 2 diabetes that have the added advantage of not promoting hyperinsulinaemia, weight gain or inducing hypoglycaemia [2], [3]. Their method of action is to block glucose entry into the kidney proximal tubular cell, a process known to be integrally involved in the development of diabetic nephropathy [4]–[6]. The resultant glycosuria does not appear to be associated with an increased risk of urinary tract infections [3], [7]. The question therefore arises as to what effect this blockade of glucose transiting through the proximal tubular cell will have on the development and progression of diabetic nephropathy and, for that reason these studies address the specific cellular effects of SGLT2inh on proximal tubule cell dysfunction.

While the traditional focus in diabetic nephropathy has been on histological changes seen in the glomerulus, it has become widely acknowledged that the changes seen in the tubulointerstitium, and in particular tubulointerstitial fibrosis, correlate more closely with deterioration in renal function [8]. In type 2 diabetes, the principal tubulointerstitial changes seen are those of proximal tubular cell (PTC) basement membrane thickening, hyperplasia and hypertrophy in early diabetes, followed by atrophy of these structures as the condition progresses [9]. Interstitial fibrosis accompanies these changes and ultimately correlates with the demise of kidney function. We have previously defined the direct effects of high glucose in mediating inflammatory and profibrotic effects in PTCs [5], [10], the specific effects of increased PTC sodium transport in early diabetes [11], [12] and the damaging effects of the hyperplastic and profibrotic cytokine TGFβ on PTC growth and function [4], [13], [14]. To this end, destructive sequelae of diabetes have been firmly linked to glucose exposure and intracellular glucose influx in PTC and it is our hypothesis that these sequelae will be moderated by blockade of the principal glucose transporters (SGLT2) in the proximal tubule of the kidney.

The sodium dependent glucose transporters (SGLT), located on the apical side of the proximal tubule cell, are able to transport glucose within the cell against a concentration gradient by transporting glucose concurrently with sodium [15]. A sodium concentration gradient is provided by the Na-K-adenosine triphosphatase (Na-K-ATPase) pump located on the basolateral membrane. Glucose is then passively transported across the basolateral side of the cell via facilitative glucose transporters (GLUT) into the interstitium. In the early segments of the proximal tubule, SGLT2 on the apical membrane is coupled with GLUT2 on the basolateral side, and together these transporters reabsorb up to 90% of filtered glucose under normoglycaemic conditions [16]. The rationale behind SGLT2inh as therapeutic agents, therefore, is that interference with the principal glucose transporters in the proximal tubule will enhance glucose excretion into the urine leading to glycosuria and thereby improved glycaemic control [15], [17]. While the ability of these agents to induce glycosuria and thereby improve blood glucose levels has been established in both animal studies and in a number of clinical trials [18]–[20], determination of their effects on the renal changes seen in the context of diabetes has not been assessed. We and others have explored the effects of SGLT2 blockade on the altered sodium handling seen in diabetes, which is known to contribute to both hypertension and glomerular hyperfiltration [12], [21] but the important question that remains to be resolved is whether SGLT2inh have additional renal benefits (beyond serum glucose lowering) by blocking excess glucose entry into the proximal tubule cells under diabetic conditions.

In our studies, we used empagliflozin (BI 10773) a novel SGLT2inh developed by Boehringer-Ingelheim. It is a very potent and selective SGLT2inh with an IC50 around 3 nM and Cmax from clinical dosing around 500 nM (personal communication with Boehringer-Ingelheim). In HEK293 cells (a human embryonic kidney cell line) overexpressing human SGLT2, empagliflozin significantly blocked ^14^C alpha methyl glucose uptake [22]. In our studies, concentrations used were 100 nM and 500 nM based on recommendations by Boehringer-Ingelheim as these doses effectively and selectively block SGLT2 without significant inhibition of SGLT1. In phase I clinical trials in patients with type 2 diabetes mellitus, once-daily empagliflozin increased urinary glucose excretion resulting in dose-proportional reductions in fasting plasma glucose and mean daily glucose levels. empagliflozin was not associated with significant hypoglycemic episodes or other clinically important adverse events and phase three clinical trials are due to report [18].

## Materials and Methods

### Cell Culture

HK2 cells, a primary human proximal tubular cell line (American Type Culture Collection), were grown in Keratinocyte Serum Free Media (KSFM) supplemented with bovine pituitary extract 20–30 µg/ml and epidermal growth factor 0.1–0.2 ng/ml (Gibco, NY, USA). Cell culture media was changed every 48 hours. These cells were grown at 37°C in a humidified 5% CO_2_ incubator and were subcultured at 50–80% confluence using 0.05% trypsin - EDTA (Gibco, NY, USA). The SGLT2inh empagliflozin was generously provided by Boehringer-Ingelheim. When 80% confluent, cells were exposed to 5 mM glucose, 30 mM D-glucose, 30 mM D-glucose plus 100 nM SGLT2inh and 30 mM D-glucose plus 500 nM SGLT2inh for up to 72 h then harvested. For the TGFβ experiments, a final concentration of 0.5 nM recombinant human TGFβ_1_ (R&D systems, MN, USA) was used instead of glucose. Recombinant high mobility group box protein 1 (ProteinOne, MD, USA) was used at a final concentration of 50 ng/ml. Preliminary viability studies were conducted using a commercial MTS assay CellTiter 96® AQ_ueous_ (Promega, WI, USA) which showed no significant cell toxicity at the concentrations used.

### Western Blot Analysis

Cells collected were 95% confluent and the cell pellet was resuspended in cell lysis buffer containing 50 mM Tris-HCl, 150 mM NaCl, 5 mM EDTA (pH 7.4), 0.5% Triton-X100, and protease inhibitors (Roche Diagnostics, Mannheim, Germany). Cell lysate was spun at 13000 rpm at 4°C for 5 minutes and stored at −20°C. Protein quantification (Bio-Rad, CA, USA) was carried out to determine the protein concentration of the cell lysate. 50–80 µg total cell protein was mixed with 6x Laemmli sample buffer containing mercaptoethanol and heated at 95°C for 10 minutes. Samples were then analyzed by sodium dodecyl sulfate polyacrylamide gel electrophoresis (SDS-PAGE) using a 10% gel and electroblotted to Hybond Nitrocellulose membranes (Amersham Pharmacia Biotech, Bucks, UK). Membranes were blocked in Tris-buffered saline containing 0.2% Tween-20 (TTBS) in 5% skim milk for 2–3 hours and then incubated overnight at 4°C with the following primary antibodies – SGLT1 and SGLT2 1:300 (Santa Cruz, CA, USA), GLUT2 1:500 (Millipore), collagen IV 1∶5000 (Abcam Ltd, Cambridge), toll-like receptor 4 (TLR4) 3 ug/ml (Invitrogen, CA,USA) in TTBS (Tris Buffered saline with 0.2% Tween) containing 5% skim milk. Membranes were washed with TTBS and incubated with horseradish peroxidase conjugated secondary antibody. Proteins were visualized using the enhanced chemiluminescence (ECL) detection system (Amersham Pharmacia Biotech, Bucks, UK). All membranes were reprobed with β actin 1∶1000 (Santa Cruz, CA, USA) and results were corrected for actin as a loading control and analysed using Image J software (Java based software program, NIH).

### Nuclear Extraction and Electrophoretic Mobility Shift Assay (EMSA)

Nuclear extracts were prepared using NucBuster™ Protein Extraction Kit (Novagen, Darmstadt, Germany) as per manufacturer’s instructions. The DIG Gel Shift Kit (Roche Applied Science, Indianapolis, US), was used in the EMSA. In brief, 25 µg of nuclear extract were incubated with 1 µg poly [d (I–C)] as the non specific competitor, 1 µg poly L-lysine in a binding buffer (100 mM Hepes, pH 7.6, 5 mM EDTA, 50 mM (NH_4_)_2_SO_4_, 5 mM DTT, Tween 20, 1% w/v, 150 mM KCl) and dig-labeled AP-1 (5′-CGC TTG ATG AGT CAG CCG GAA-3′) and dig-labeled NF-κB (5′-AGT TGA GGG GAC TTT CCC AGG C-3′) consensus oligonucleotide (Promega, WI, USA) for 30 minutes at room temperature. The reaction mixture was electrophoresed through 6% polyacrylamide gels, transferred onto nylon positively charged membrane (Roche Applied Science, Indianapolis, USA) and then crosslinked using an UV-transilluminator for 3 minutes. The membrane was subjected to immunological detection using anti-Digoxigenin-AP conjugate and chemiluminescence. Results were analyzed using Image J software and shift bands were measured.

### Interleukin 6 (IL6) ELISA

HK-2 cells were seeded at 15×10^4^ cells/well in a 24 well plate. Cells were treated for 48 h and supernatants were collected, centrifuged at 13000 rpm for 5 minutes and stored at –20°C until IL6 levels were determined with an immunoassay kit assay (Invitrogen, CA, USA) as per manufacturer’s instructions and read using a microplate reader at 450 nm. Cell lysate protein concentration was determined using Bio-Rad Protein assay and IL6 levels were corrected for protein content per well.

### Chromatin Immunoprecipitation Assay

Chromatin immunoprecipitation (ChIP) assay was performed using Pierce Agarose ChIP kit (Thermo Fisher Scientific, IL USA) according to the manufacturer’s instructions. In brief, 2×10^6^ cells were cross-linked in 1% formaldehyde after exposure to control media and 0.5 ng/ml TGFβ for 72 hours. Cells were then lysed and chromatin was sheared using micronuclease digestion 200- to 1000-bp DNA fragments. Input DNA was stored for subsequent quantitation. Remaining sheared DNA was incubated with anti-phosphosmad3 antibody (Cell Signaling Technology). Normal Rabbit IgG and anti-RNA polymerase II antibody were used as negative and positive control respectively. The crosslink of immunoprecipitated protein-DNA samples were then reversed, and DNA samples were purified using spin columns. Phosphosmad3 binding sites to the SGLT2 promoter region were analysed using quantitative real time PCR with SYBR green (ABI Prism 7900 HT). The primers used were forward primer 5′-G T C T A A G G C G C A G T C T G A G G-3′ and reverse primer 5′-C T G C A C G C T T G G A G T A G A T G-3′). PCR conditions were 95°C for 5 min and then 40 cycles at 94°C (40 s), 50°C (40 s) and 72°C (90 s). Samples were done in triplicate and data is expressed as % of input values. The amplified PCR product was analysed on a 1.2%agarose gel.

### Statistical Analysis

All the results are expressed as normalised results and are a percentage of the mean±standard error of control values. Experiments were performed at least in triplicate or as detailed in the text with *n* reflecting the number of separate experiments. Statistical comparisons between groups were made by analysis of variance (ANOVA) or unpaired t-tests where appropriate. Analyses were performed using the software package, Statview version 5.0 (Abacus Concepts Inc., Berkley, CA, USA). P values <0.05 considered significant.

## Results

### SGLT2 Expression is not Regulated by High Glucose but is Increased with TGFβ_1_ via Phosphosmad3 Signaling

**Figure 1:**
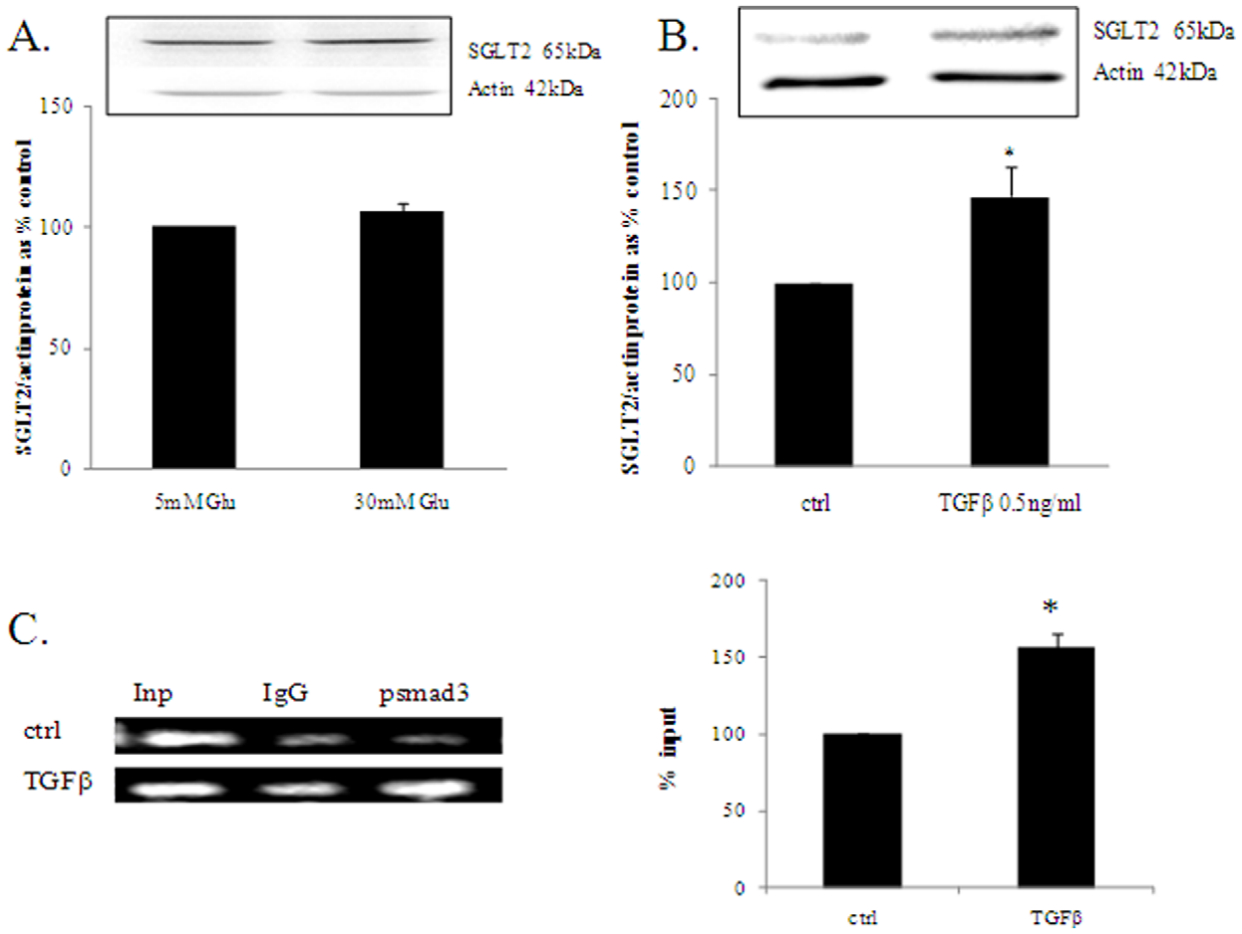
The regulation of SGLT2. SGLT2 expression is not regulated by high glucose (A) but is increased with TGFβ_1_(B) in HK2 cells after 48 hours (B). Chromatin immunoprecipitation assay showed significantly increased binding of phosphosmad3 to the relevant binding site in the promoter region of the SGLT2 gene with TGFB treatment compared to control. Results were normalized to input DNA and expressed as % input of 3 separate experiments where input represents the amount of chromatin used. Amplified PCR products were also analysed on a agarose gel (C). HK2 cells were incubated with 5 mM (ctrl), 30 mM high glucose, 0.5 ng/ml TGFβ1 and the SGLT2 inhibitor empagliflozin at 100 nM and 500 nM final concentration. SGLT2 protein expression was assessed using western blot. Normalized results are expressed as mean±SEM, n = 4–6. * p<0.05 vs control.

SGLT2 is the predominant glucose cotransporter in human kidney proximal tubular cells. In order to determine whether SGLT2 expression is regulated by high glucose in HK2 cells, they were incubated with 30 mM glucose for 24 h, 48 h and 72 h. We found that SGLT2 expression did not change with high glucose at 48 h as shown in Figure 1A. Experiments were repeated at 24 h and 72 h to ensure that we are not missing the right time point and this also showed no change (data not shown). This result implies that high glucose does not regulate SGLT2 expression at a protein level which would suggest that acute hyperglycaemia itself is not sufficient to potentiate further glucose reabsorption. High glucose in itself increases TGFβ_1,_ a prominent profibrotic cytokine responsible for laying down extracellular matrix in diabetic nephropathy. We have previously shown that HK2 cells exposed to high glucose increases secreted TGFβ_1_ *in vitro* [5]. Motif searching identified Smad 3 and 4 binding sites in the promoter region of SGLT2, which, together suggest that SGLT2 expression is under the direct control of the classic TGFβ signalling pathway. Hence we looked at whether TGFβ_1_ regulated SGLT2 expression by exposing HK2 cells to recombinant human TGFβ_1_ for 72 h. We found that TGFβ1 increased SGLT2 protein expression to 146.7±16.5% of control after 72 h as shown in Figure 1B (p<0.05). Chromatin immunoprecipitation studies with real time PCR confirmed significantly increased binding of phosphorylated smad3 to the promoter region of the SGLT2 gene with TGFβ_1_ treated cells showing a % input of 156.3±9.3 compared to control (p<0.05). This is also represented visually on a agarose gel as shown in Figure 1C.

### SGLT1 and GLUT2 Expression is not Altered with High Glucose and SGLT2 Inhibition

**Figure 2:**
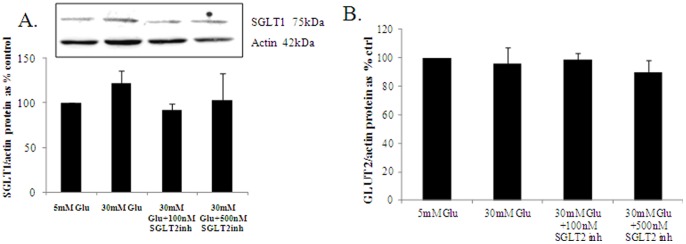
The regulation of SGLT1. SGLT1 expression is not significantly altered with 30 mM high glucose and SGLT2 inhibition after 72 hours (A). GLUT2 expression is not altered with 30 mM high glucose and SGLT2 inhibition (B). HK2 cells were incubated with 5 mM (ctrl), 30 mM high glucose, and the SGLT2 inhibitor empagliflozin at 100 nM and 500 nM final concentration. SGLT1 protein expression was assessed using western blot. Normalized results are expressed as mean±SEM, n = 4–6.

Glucose reabsorption in the human proximal tubular cell is affected by other glucose transporters. SGLT2, situated in the S1 segment, is a low-affinity high-capacity transporter reabsorbing up to 90% of filtered glucose. SGLT1, situated in the S3 segment, is a high-affinity low-capacity transporter reabsorbing the remaining 10% [15]. GLUT2 is a facilitative transporter located at the basolateral membrane. So, it is important to measure SGLT1 and GLUT2 expression (the minor glucose transporters) in order to ascertain whether high glucose or SGLT2inh is causing a compensatory increase in these protein expression as any compensatory increase in expression of SGLT1 or GLUT2 may negate the effect of SGLT2 inhibition by allowing glucose to enter the cells. We found that SGLT1 and GLUT2 protein expression was not significantly altered with high glucose or the SGLT2inh as shown in Figure 2A and B. This implies that blocking SGLT2 with empagliflozin is unlikely to causes a compensatory increase in the other glucose transporter on HK2 cells in our experiments.

### SGLT2inh Reverses High Glucose Induced TLR4 Expression

**Figure 3:**
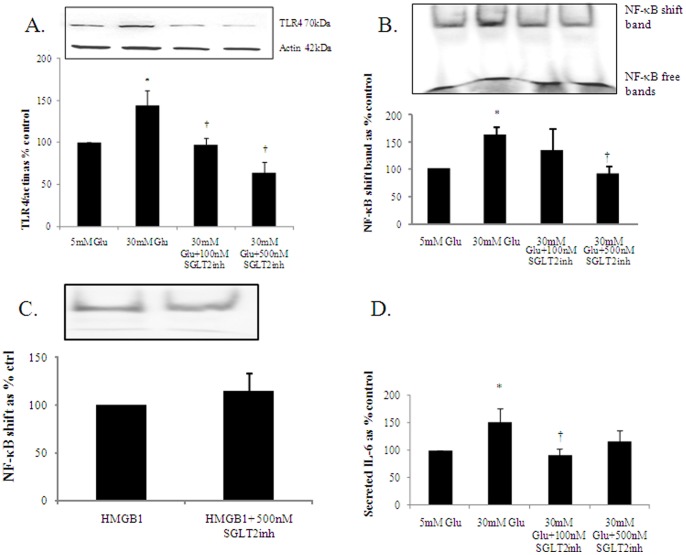
The effect of SGLT2inh on inflammatory markers. SGLT2inh significantly reverses high glucose induced TLR4 expression at 24 hours (A) and NF-κB binding at 72 hours (B). This was specific to blockade of glucose entry into the cell as another stimulus of NF-κB binding like HMGB1 was not affected by SGLT2 inhibition (C). SGLT2inh also reduced high glucose induced IL-6 secretion (D). HK2 cells were exposed to 5 mM (ctrl), 30 mM high glucose, 50 ng/ml recombinant HMGB1 and the SGLT2 inhibitor empagliflozin at 100 nM and 500 nM final concentration. TLR4 expression was assessed with western blot and NF-κB binding was measured using EMSA. For the HMGB1 experiments, cells were pretreated with the SGLT2 inhibitor for 24 hours then exposed to recombinant HMGB1 for 2 hours. IL-6 was measured in the supernatant using a commercial ELISA kit Normalized results are expressed as mean±SEM, n = 5.* p<0.05 vs control; † p<0.05 versus 30 mM Glu.

TLR4 is a ligand activated membrane bound receptor and is involved in NF-κB mediated inflammation After 24 hours, high glucose induced TLR4 expression to 144.1±18.1% of control (p<0.05). The SGLT2inh at both concentrations significantly inhibited high glucose induced TLR4 expression to 97.2±8.2% and 64.4±12.6% respectively, p<0.05 as shown in Figure 3A. This would suggest that the increase in glucose induced TLR4 is likely to be due to intracellular glucose entry rather than glucose acting as a TLR4 ligand as it was reversed with empagliflozin.

### SGLT2inh Reverses High Glucose (but not High Mobility Group Box Protein 1) Induced NF-κB Binding

After exposing HK2 cells to high glucose for 72 h, high glucose induced NF-κB binding increase as expected to 161.1±16.4% of control (p<0.05). Empagliflozin at a final concentration of 500 nM significantly inhibited high glucose induced NF-κB binding to 91.7±14.9% of control, p<0.05. This is shown in figure 3B. In order to determine that these changes are specific to glucose, we repeated these experiments using recombinant high mobility group box protein 1 (HMGB1), as we have previously shown that it is a potent stimulus of NF-κB binding in these cells [23]. We demonstrate that in the absence of high glucose empagliflozin does not reduce HMGB1 induced NF-κB binding, reflecting the specificity of glucose blockade. This is shown in Figure 3C.

### SGLT2inh Reduces High Glucose Induced IL-6 Secretion

IL-6 is a secreted proinflammatory cytokine and after 48 h, high glucose induced IL-6 secretion to 151.0±26.1% of control, p<0.05. Empagliflozin at 100 nM final concentration reduced high glucose induced secreted IL-6 to 92.0±11.7% of control, p<0.05. Similarly, at 500 nM there was a reduction of 1L-6 to 116.5±19.6% which was comparable to control values as shown in Figure 3D.

### SGLT2inh Reverses High Glucose Induced AP-1 Binding

**Figure 4:**
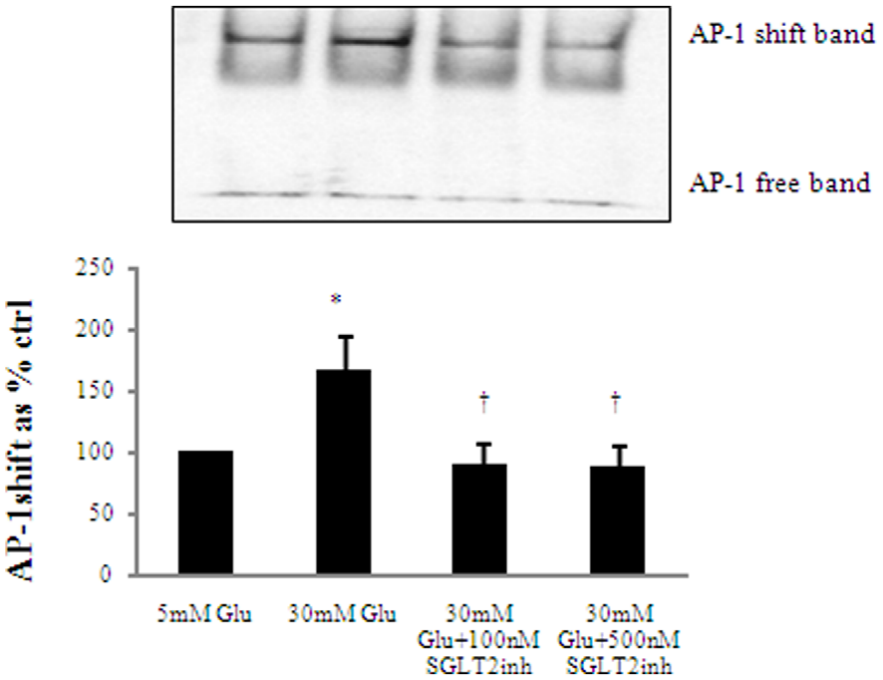
The effect of SGLT2inh on AP-1. SGLT2inh significantly reverses high glucose induced AP-1 binding. To assess the level of AP-1 binding, HK2 cells were incubated for up to 72 h with 5 mM (ctrl) media, 30 mM high glucose and SGLT2inh at 100 nM and 500 nM. AP-1 binding was assessed using EMSA**.** High glucose induced AP-1 binding and the SGLT2inh at both concentrations significantly inhibited this increase. Normalized results are expressed as mean±SEM, n = 3.* p<0.05 versus control; † p<0.05 versus 30 mM Glu.

Both NF-κB and AP-1 are key transcription factors mediating the fibrotic and inflammatory pathways in HK2 cells exposed to high glucose [5], [10] Similar to NF-κB, 72 h of exposure to high glucose induced AP-1 binding as expected to 166.7±27.6% of control, p<0.05. Empagliflozin at a final concentration of 100 nM and 500 nM significantly inhibited high glucose induced AP-1 binding to 91.7±14.9% of control and 87.9±18.5% of control respectively, p<0.05 as shown in Figure 4. This would imply that intracellular glucose levels determine the activity of both these transcription factors.

### SGLT2inh Reverses High Glucose Induced CIV Expression

**Figure 5:**
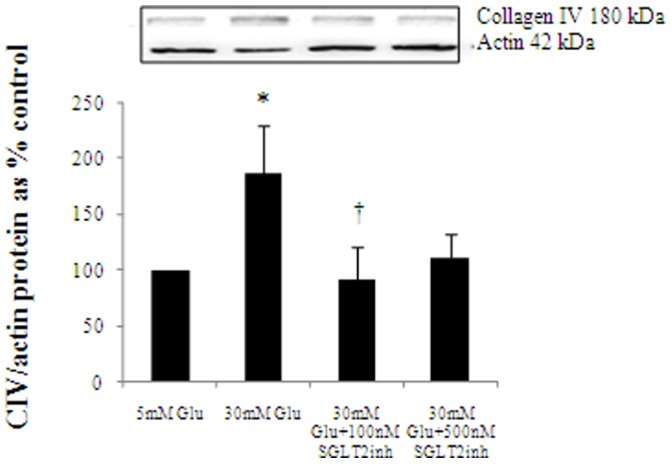
The effect of SGLT2inh on CIV. SGLT2inh reverses high glucose induced CIV expression. To assess the level of CIV expression, HK2 cells were incubated for up to 48 h with 5 mM (ctrl) media, 30 mM high glucose and SGLT2inh at 100 nM and 500 nM. High glucose induced CIV expression and the SGLT2inh at 100 nM significantly inhibited this increase. Although SGLT2inh at 500 nM reduced CIV expression, it did not reach statistical significance. Normalized results are expressed as mean±SEM, n = 4. * p<0.05 versus control; † p<0.05 versus 30 mM Glu.

CIV is a basement membrane matrix protein which is induced by high glucose in HK2 cells. When cells were incubated for 48 h, high glucose induced CIV expression to 185.6±42.8% of control, p<0.05. Empagliflozin at 100 nM final concentration reduced high glucose induced CIV expression to 91.8±29.8% of control, P<0.05. At 500 nM, empagliflozin reduced CIV expression to 110.8±21.0% which was comparable to control values. This is shown in Figure 5.

## Discussion

SGLT2 inhibitors lower serum glucose in type 2 diabetes mellitus but blocking the reabsorption of glucose which occurs in the kidney proximal tubular cells via SGLT2 (the main transporter). This begs the question – does this protect the proximal tubular cells from glycotoxicity? Our studies initially looked at whether high glucose altered the expression of the SGLTs in HK2 cells, a human kidney proximal tubule cell line. The findings suggest that there is no regulation of these glucose transporters in response to a high glucose milieu, as high glucose did not alter the protein expression of SGLT1 or SGLT2. Although SGLT1 is not the main glucose transporter in the early proximal tubule, we measured its expression in the context of SGLT2inh to assess whether there was any compensatory increase in glucose transport via SGLT1. We presume that this is unlikely given the lack of increase in SGLT1 expression with high glucose when combined with a SGLT2inh.

Interestingly, recombinant human TGFβ_1_ upregulated SGLT2 at a protein level (in normal glucose conditions). TGFβ is recognised as the prototypical fibrogenic and hypertrophic cytokine that has been found to stimulate PTCs to produce key extracellular matrix molecules including type I collagen, type IV collagen, fibronectin and laminin [24] and is intrinsic to the development of diabetic nephropathy. Virtually all of the molecular mediators and intracellular signalling pathways that have been identified in diabetic nephropathy, have also been found to stimulate renal TGFβ activity as an intermediary step including: high glucose concentration [25], reactive oxygen species [26], angiotensin II [27], exposure to advanced glycation end-products [28], protein kinase C activation [29] and endothelin [30]. The classical TGFβ signalling pathway commences when TGFβ dimers bind to a type II receptor which recruits and phosphorylates a type I receptor, followed by the recruitment and phosphorylation of Smad3. Smad3 then binds to Smad4 to form a heterodimeric complex that acts as a transcription factor for various genes. Our preliminary data has shown that HK2 cells exposed to TGFβ_1_ show an increase in SGLT2 expression and motif searching has identified Smad 3 and 4 binding sites in the promoter region of SGLT2 and this was confirmed by the chromatin immunoprecipitation studies which, together suggest that SGLT2 expression is under the direct control of the classic TGFβ signalling pathway via smad3. This is a novel finding linking TGFβ action to SGLT2 expression in a potentially vicious cycle whereby TGFβ increases the expression of SGLT2, allowing for an increase in intracellular glucose leading to further TGFβ production.

We have also uniquely demonstrated that the SGLT2 inhibitor empagliflozin caused a reduction in high glucose induced TLR4, CIV and IL6 expression in addition to a suppression of high glucose induced DNA binding activity of NF-κB and AP-1. TLRs are germline-encoded innate immune receptors expressed in leukocytes and kidney cells, and are involved in inflammation upon activation by exogenous (pathogen derived lipopolysaccharides) or endogenous (tissue derived) ligands. We have shown that endogenous ligands to TLR4 such as fibronectin, heat shock protein 70 (HPS70) and high mobility group box 1 (HMGB1) are upregulated in the presence of high glucose [5], [10]. The TLR4 signaling pathway converges at NF-κB an important immunomodulatory protein. As these factors are inextricably linked to the inflammatory and fibrotic process in diabetic nephropathy, it would suggest that SGLT2 inhibitors may be useful in limiting glucose induced renal inflammation above and beyond its serum glucose lowering effects. A consistent overall effect was seen whereby SGLT2 inhibitors alleviated the damaging effects of high glucose but these occurred at different concentrations of the inhibitor, reflecting presumed differences in intracellular glucose concentrations at which these effects occur. With respect to NF-κB, these results are likely because of reductions in intracellular glycotoxicity rather than non specific effects of empagliflozin as NF-κB binding was not reduced when another stimulus like HMGB1 was used. This would suggest that injurious pathways unrelated to glycotoxity may not be altered by SGLT2 inhibition. Hence these *in vitro* findings may or may not be predictive of *in vivo* findings. *In vivo*, although SGLT2 inhibitors may protect the proximal tubular cells from glycotoxicity, the other parts of the kidney like the vasculature and the glomeruli would still be subjected to existing serum glucose levels.

In conclusion, our studies are the first to provide *in vitro* evidence in human proximal tubular cells to suggest that the SGLT2inh, empagliflozin, is able to limit high glucose induced inflammatory and fibrotic markers most likely due to blocking glucose entry into the cell and that TGFβ_1_ (but not high glucose) regulates SGLT2 expression via the classical signalling pathway involving phosphorylated smad3. Clinical studies will be required to ascertain whether SGLT2 inhibitors offer additional renal protection compared to other oral hypoglycaemic agents used to treat type 2 diabetes mellitus. If additional renoprotection above and beyond plasma glucose lowering is evident, then SGLT2 inhibitors may be the oral hypoglycaemic agent of choice given its other favourable features in regard to weight and adverse hypoglycaemia.
